# Exploring the RNA Gap for Improving Diagnostic Yield in Primary Immunodeficiencies

**DOI:** 10.3389/fgene.2019.01204

**Published:** 2019-12-11

**Authors:** Jed J. Lye, Anthony Williams, Diana Baralle

**Affiliations:** ^1^University of Southampton Medical School, University of Southampton, Southampton, United Kingdom; ^2^Wessex Investigational Sciences Hub Laboratory (WISH Lab), Faculty of Medicine, University of Southampton, Southampton, United Kingdom; ^3^Faculty of Medicine, Highfield Campus, University of Southampton, Southampton, United Kingdom

**Keywords:** primary immunodeficiency disorders, clinical diagnostics, RNASeq, RNA, RNAseq analysis

## Abstract

Challenges in diagnosing primary immunodeficiency are numerous and diverse, with current whole-exome and whole-genome sequencing approaches only able to reach a molecular diagnosis in 25–60% of cases. We assess these problems and discuss how RNA-focused analysis has expanded and improved in recent years and may now be utilized to gain an unparalleled insight into cellular immunology. We review how investigation into RNA biology can give information regarding the differential expression, monoallelic expression, and alternative splicing—which have important roles in immune regulation and function. We show how this information can inform bioinformatic analysis pipelines and aid in the variant filtering process, expediting the identification of causal variants—especially those affecting splicing—and enhance overall diagnostic ability. We also demonstrate the challenges, which remain in the design of this type of investigation, regarding technological limitation and biological considerations and suggest potential directions for the clinical applications.

## Introduction

Primary immunodeficiency disorders (PID) result from altered, poor, or absent function in one or more components of the immune system, rendering the affected individuals with increased susceptibility to immune-related ailments including increased frequency and severity of infection, autoimmunity, aberrant inflammation, and malignancy (McCusker et al., 2018). The understanding of the genetic heterogeneity of PID has expanded greatly over the last decade, now encompassing a list of over 350 distinct disorders arising from at least 344 gene defects, demonstrative of the complexity of the immune system (Bousfiha et al., 2018; Picard et al., 2018). This plethora of genetic causes has brought about a need to categorize the disorders for expedited diagnosis and treatment protocols. Some broader methods simply classify the disorders into groups of innate and adaptive immunity linked to the clinical phenotype (McCusker and Warrington, 2011). The Inborn Errors of Immunity Committee (previously the International Union of Immunological Societies PID Expert Committee) has now devised a precise and useful system, which classifies disorders by the immunological pathway affected. In addition, it now has corresponding phenotypical classification systems for clinicians at the bedside to help identify the disorders. These briefly comprise nine categories: immunodeficiencies affecting cellular and humoral immunity, combined immunodeficiency disorder (CID) with associated or syndromic features, predominantly antibody deficiencies, diseases of immune dysregulation, congenital defects of phagocyte, defects in intrinsic and innate immunity, auto-inflammatory disorders, complement deficiencies, and phenocopies of PID (Bousfiha et al., 2018).

The most common form of PID is selective immunoglobulin A deficiency, which is usually typically asymptomatic but can manifest with a variety of clinical presentations including coeliac disease, type 1 diabetes mellitus, and increased infections. With an estimated prevalence of 1 in 300–500 persons (Boyle and Buckley, 2007), and while individually rare (>1 in 2,000), the remaining disorders considered in the wider scope of PID together represent a significant burden on the health and economy of a nation. Current diagnostic levels suggest an incidence of 5.90/100,000 (Shillitoe et al., 2018); however, underdiagnsosis of PID may mean the true incidence is as high as 1:250 (Europe PIPDDfOCi.).

The importance of early diagnosis In PID cases is high, with relation to both the patient’s qualitative experience and the economic cost to healthcare services. Sources vary in cost analysis of undiagnosed PID. Some say that while a diagnosed US patient costs healthcare services over US$250,000 per annum, largely due to treatment costs, an early diagnosis of the disorder can save as much as US$6,500 per patient, per annum (Abolhassani et al., 2015). An alternate source suggests an undiagnosed patient might cost the healthcare system US$102,552 annually. Once diagnosed, these costs may drop by as much as US$79,942 (Condino-Neto and Espinosa-Rosales, 2018). In a patient survey, 45% of patients reported a diagnostic wait time of between 1 and 6 years; around 1/6th reported waiting 10–20 years. Other key findings of the same survey confirmed undiagnosed patients bring about a dramatically increased burden on National Health Service (NHS) resources (). Identification of the precise molecular origins for each patient’s case of PID leads to improved patient care (Walter et al., 2016) and improved prognosis. The importance of correct genetic cause for a PID phenotype is demonstrated by the different treatment preferences which exist for conditions which may present with similar clinical phenotypes (Heimall et al., 2012). Precision therapeutic diagnostics can help to achieve this in part, by allowing targeted intervention to the specific molecular causes (Bonilla et al., 2015; Lenardo et al., 2016; Ramakrishnan et al., 2016).

### Diagnostic Challenges in PID

Challenges in diagnosing primary immunodeficiency are numerous and diverse. Studies which correlate the phenotype and genotype have been useful in diagnostics, developing an understanding of various PID disorders (Fischer, 1993). Additionally, these correlation studies have been useful for deconvoluting the pleiotropic nature of the involved genes, through which a single variant can bring about a variety of clinical phenotypes (Meyts et al., 2016). However, the development of a universal diagnostic pipeline for PID is hindered by the heterogeneity in the presentation of disease, even among patients with what appears to be the same pathogenic genetic variant (Richardson et al., 2018). Conversely, a number of genotypes can bring about even the most well-characterized phenotype (Meyts et al., 2016). Once a clinical diagnosis of PID is suspected, mainly based upon a compatible phenotype, a family history is usually taken and a number of subsequent laboratory tests performed to confirm the type of immune mechanism affected (Hernandez-Trujillo and Ballow, 2015). With the emergence of targeted sequencing of larger PID gene panels, clinical exomes, and complete exomes through short-read next-generation sequencing technologies, the inclusion of genetic testing within a PID diagnostic workup has become more widespread. This approach to both adult- and paediatric-onset disease has consolidated the importance of protein-based functional immune testing (cytokines, antibodies, etc.) for characterizing the nature of the phenotypic presentation, but furthermore to evaluate candidate genetic variants in such pathways that have been identified through parallel germline DNA testing.

DNA sequencing-based genetic testing is used where possible, as it provides the best diagnostic capability of existing clinically adapted methods (Condino-Neto and Espinosa-Rosales, 2018). Whole-exome sequencing (WES) has the highest success rate of the clinically adapted diagnostic methods (Gilissen et al., 2011; Boycott et al., 2017), which it achieves despite the exome comprising only ∼2% of the human genome (Bamshad et al., 2011).This is in part due to 85% of currently annotated variants existing within the transcribed portion of the genome (Majewski et al., 2011). It has been hypothesized that this focus had likely led to the underestimation of the contribution to disease of non-coding variants (Kremer et al., 2018).

Due to the improvement that WES and whole-genome sequencing (WGS) bring to diagnostics, researchers are calling for universal molecular gene testing for the diagnosis of primary immune deficiencies (Heimall, 2019). Evidence from existing literature, however, suggests that even this may be inadequate; currently, WES and WGS are only able to produce reliable diagnosis in 25–60% of cases (Yang et al., 2013; Moens et al., 2014; Yang et al., 2014; Taylor et al., 2015; Meyts et al., 2016; Stray-Pedersen et al., 2017). Although many countries have undertaken whole-genome sequencing projects to evaluate this approach (Philippidis, 2018), the development of WGS as a clinically validated routine testing modality is still in its infancy. Within the UK’s 100,000 Genomes Project, PID was accepted as an indication for inclusion, and plans to incorporate WGS for PID into routine clinical pathways have been approved following the transition phase of 100K Project to WGS sequencing in routine NHS care across England.

Formal confirmed genetic diagnosis of PID relies heavily on existing knowledge pertaining to consequences of the variants in the genes of relevance to the presenting phenotype and assumed mechanism of disease resulting from such variants in a dominant or recessive manner genomic sequence (Rae et al., 2018). The key to this task is the ability of bioinformatics tools to predict the significance of such variants. WES delivers around 20,000–23,000 variants per individual, and WGS produces 3–5 million per individual (Kremer et al., 2018), which makes the task of identifying a Mendelian disease variant vanishingly unlikely without a series of bioinformatics filters. Problems with the WGS/WES sequencing diagnostic methods arise when no variant, identified through a patient’s genome sequencing, can be reliably linked to the clinical presentation and cytological/molecular manifestation of the disorder. Failure to identify a definitive molecular cause occurs in about 70–75% of Mendelian conditions, according to a 2018 meta-analysis (Schwarze et al., 2018), mirrored by examples from PID (Gallo et al., 2016). The types of variants which are not always identified by current next-generation sequencing (NGS) approaches include exonic variants of unknown significance, variants in intronic and intergenic non-coding DNA (Scacheri and Scacheri, 2015), variants in the *cis*-acting regulatory elements of transcription (Bryois et al., 2014) imprinting disorders, and repeat expansions (Kremer et al., 2018).

Conventional clinical diagnostics, utilizing human phenotype ontology for integration of cases into specific diagnostic groups, and traditional genetic sequencing methods for diagnostics are still currently inadequate. While proteomic diagnostic methods are in development, they exist at a relatively early stage of development and can miss the potentially valuable RNA regulatory phenomena.

### Variants Affecting Differential Expression

Identification of definitive disease-causing mutations is confounded in some cases by expression levels being modulated by variants occurring in non-coding segments and those hiding in plain sight in genes not currently understood to be linked to the disease or phenotype. Often, these can be lost during the filtering process because of a lack of integrative understanding or supporting evidence (Thormann et al., 2019).

These expression quantitative trait loci (eQTLs) elicit a powerful, sometimes synergistic effect on the expression of a large number of genes. Single-nucleotide polymorphisms (SNPs) on eQTLs affect the transcriptional level of other RNAs, modifying protein expression and causing phenotypic changes to the abilities and behaviors of cells in some immunological cases (Fairfax et al., 2014). These eQTLs individually explain a fraction of the genetic expression of specific genes. The vast majority do not exist in the coding regions of genes and are predicted to be involved in gene regulation (Casamassimi et al., 2017). It is now understood that these eQTLs have a more pronounced effect on immune regulation than the effects of age and sex, and more interestingly exclusive effects only observable during immune stimulation have been identified for some of these eQTL variants (Piasecka et al., 2018). Epigenomic studies have helped to highlight the *cis*-regulatory nature of some non-coding regions of the genome. These suggest that the enrichment of disease-risk variants in cell-specific regulatory sequences is indicative of their cell type and contextual effects (Roadmap Epigenomics et al., 2015). Large-scale investigation into the association between genetic variants and expression of genes in a tissue-specific manner (including whole blood) was carried out by the Genotype—Tissue Expression Consortium (The Genotype-Tissue (GTEX), 2015). This research did not extend to immune tissues specifically, although links between immune cell-specific gene expression levels and eQTLs have been investigated by the DICE Project (Database of Immune Cell Expression, Expression Quantitative Trait Loci and Epigenomics) (Schmiedel et al., 2018). The researchers on this project were able to positively identify a range of *cis*-eQTLs for 12,254 genes, demonstrative of the high abundance of these sites. Interestingly, many of these eQTLs had effects which were cell type-specific. The identification of these sites and interrogation for the existence of variants will likely play a crucial role in explaining the changes in expression of key genes which lead to PID.

### The Role of RNA Splicing in the Immune System and PID

Alternative splicing is the method through which the cell can produce an array of transcript isoforms derived from a single gene or multiple genes spliced together (Ward and Cooper, 2010). Introns are spliced out and exons are either ligated through transesterification reaction or, in many cases, spliced out in different combinations, leaving the remaining exons to form a mature mRNA (Ward and Cooper, 2010).

Deep surveying on alternative splicing has shown that 95% of genes which contain multiple exons undergo alternative splicing, and even when only considering moderate to high abundance events, there are reportedly 100,000 individual splicing events in major tissues (Pan et al., 2008).

Alternative splicing occurs both co-transcriptionally and post-transcriptionally, and the action of transcription factors as well as splicing factors regulates and influences splicing events in some of the most crucial mechanisms of the adaptive immune system (Heyd et al., 2006; Orvain et al., 2008; Alkhatib et al., 2012). Important examples include RNA-polymerase II as a facilitator of splicing factor recruitment (Bentley, 2014), the alternative splicing of CD45 which is necessary for the production of a range of tyrosine phosphatases, imperative for the diverse set of lineage and stage-specific receptor signal transduction thresholds in immune tissues (Zikherman and Weiss, 2008), and FOXO1-induced Ikaros splicing, essential for the recombination of immunoglobulin genes. FOXO1 is a transcription factor which, through its effects on alternative splicing, allows the immune system to produce its diverse range of antibodies/immunoglobulins (Reynaud et al., 2008).

Activation of lymphocytes is a key component of the adaptive immune response to pathogens (Bonilla and Oettgen, 2010). Part of the central activation of these cells is the degradation of IκBα and release of NF-κB, which translocates to the nucleus to initiate maturation and activation of the cell. The “CBM” complex, which brings about the degradation of IκBα, is formed by *CARMA1*, *BCL10*, and *MALT1* (Oeckinghaus et al., 2007). *MALT1*, a crucial component of this complex, undergoes alternative splicing of EXON 7 to produce mRNA isoforms with a differential function. The activation strength of CD4^+^ T cells is mediated by the relative abundance of the alternatively spliced isoforms of MALT1, which is in part controlled by the molarity of phosphorylated splicing factor hnRNPU in the nucleus (Meininger et al., 2016). Alternative splicing, then, is a key component of the normally functioning immune system, and perturbations in canonical function can likely lead to pathology.

### Variants Affecting Alternative Splicing

**Figure 1:**
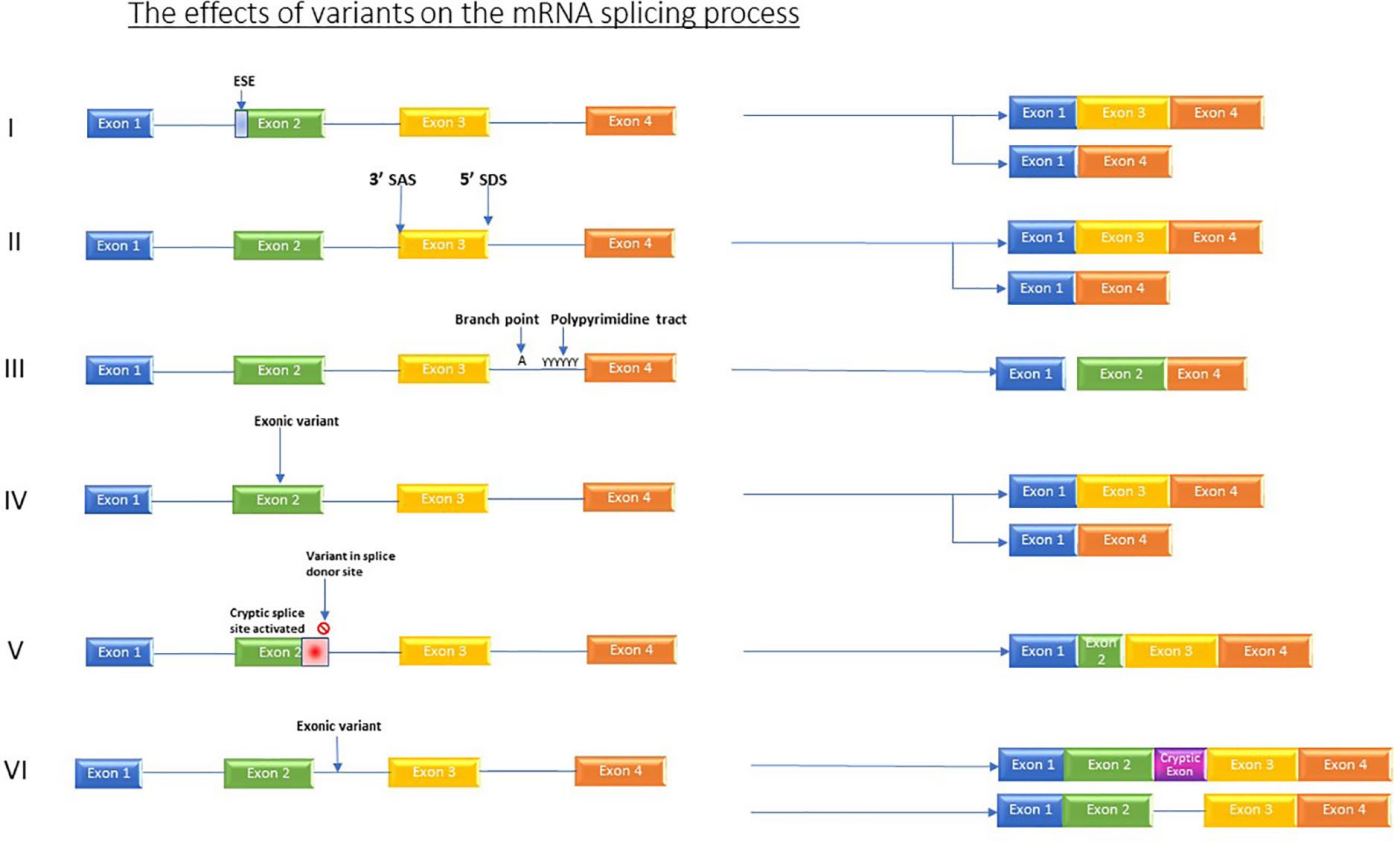
Shows the various effects which variants can have on the splicing process. I) Variants in regulator elements such as exon splicing enhancers resulting in for example, exon skipping. II) Variant in splice acceptor OR splice donor site causes skipping of one or more exons. III) Branch point and Poly pyrimidine tract sequence variants causing exon skipping. IV) Exonic variants causing exon skipping. V) Variant in splice donor site induces activation of an alternative, cryptic splice donor site in exon. VI) Intronic variants producing new cryptic exon or retained intron.

The impact of mutations that affect RNA processing/splicing is currently providing a diagnostic revolution. Variants which affect splicing either occur in active splice sites, those which occur in regulatory elements, and those which occur in intronic or intergenic regions (Grodecká et al., 2017) see **Figure 1**.

Studies comparing variants affecting splicing in PID have determined that the variants which directly influence splice sites are more robustly linked to disease phenotypes than those which effect splicing regulatory elements (Grodecká et al., 2017). *Cis*-mutations in the genome can affect splicing though altering the splice site recognition or altering exon splicing enhancer or silencer sites (Ward and Cooper, 2010). Splice sites usually comprise GT and AG dinucleotides at 5′ and 3′ sites, respectively. If a variant changes this sequence, or causes another one to appear, it can affect the ability of the splicing machinery to detect the canonical splice site (Krawczak et al., 1992; Krawczak et al., 2007). Additionally, mutations in *trans*-acting splice factors—the splicing machinery of the cell—can also bring about disease by preventing these factors from performing their function of generating the required isoforms (Ward and Cooper, 2010), although these are not covered in this review.

Due to the impact of these findings, interest in the detection of splice-altering variants and activated cryptic splice site has spurred on the development of a number of *in silico* tools for prediction of splice site usage (Grodecká et al., 2017; Ohno et al., 2018). Unfortunately, these tools are often unable to discern the resulting transcripts exon use patterns (Jaganathan et al., 2019), and while their predictive ability can be enhanced by other orthogonal investigations such as mini-gene assays (Grodecká et al., 2017), the multiple facets of splicing control involve more than just the sequence of the splice site in question, as evidenced by the temporal and spatial differences in splicing patterns. Briefly, these include the activation of other splice sites within the gene, splicing quantitative trait loci, the relative abundance, phosphorylation status, and localization of different and often competing *trans*-acting factors (Wang et al., 2015).

Further complicating this process, seemingly benign, synonymous exonic variants can disrupt splicing to cause disease. Using RNA sequencing (RNASeq) to complement genomic sequencing, Cummings et al. evidenced this in the *POMGNT1* and *RYR1* genes, finding variants which were demonstrated to be causative of Mendelian diseases in muscle (Cummings et al., 2017). Part of the normal filtering process which many bioinformaticians adopt is to filter out synonymous variants very early on, but investigation using deep learning has led to the understanding that between 9% and 11% of rare genetic disorders are caused by synonymous or intronic splice-altering mutations (Jaganathan et al., 2019). Indeed, much as gene expression can be influenced by multiple loci, so too can multiple loci contribute to the occurrence of splicing events. These loci are appropriately termed splicing quantitative trait loci (sQTLs) (Jia et al., 2015). Analysis of sQTLs has been improved by RNASeq methodologies, but remains a difficult challenge as the isoform expression has to be estimated using statistical methods (Patro et al., 2017). These sQTLs are not necessarily in close proximity to the splice junction. Characterization of these sites in humans has shown SNPs demonstrating tangible sQTL activity at 100 kb from the relative splice site (Takata et al., 2017).

Non-protein-coding genes are a significant source of disease-causing variation (Scacheri and Scacheri, 2015). Examples within the PID research and diagnosis space include a recently discovered variant occurring in coding regions for genes comprising RNA components of the minor spliceosome, which is used for the splicing of at least one exon in ∼800 genes (Turunen et al., 2013). Specifically, the non-coding gene *RNU4ATAC* that produces a small nuclear RNA (snRNA) termed U4atac was discovered to cause Roifman syndrome (Merico et al., 2015; Heremans et al., 2018) by preventing canonical minor intron splicing. Compound heterozygous variants were first discovered in an affected family after traditional filtering methods had not detected viable variants; the link was confirmed by the detection of intron retention during curated splicing analysis of RNASeq data (Merico et al., 2015).

The importance of alternative splicing in the immune system has been further demonstrated in mouse models. The ImmGen Project was set up specifically to investigate gene expression and regulation in mice using microarray profiling. It found that, in mice, around 60% of genes are expressed as multiple isoforms in T or B cells, and 70% of these had an impact on the lineage differentiation (Ergun et al., 2013). Compound heterozygous mutations *in MALT1*, mentioned earlier, which is heavily implicated in the activation of T cells, have been shown to bring about profound combined immunodeficiency. One of these variants was indeed a splice site acceptor change from the consensus AG to GG, identified by whole-exome sequencing (Punwani et al., 2015).

To further complicate the already complex nexus of control mechanisms contributing to PID, a range of epigenetic mechanisms leading to primary Immunodeficiencies have been observed and reviewed (Campos-Sanchez et al., 2019). In principle, the majority of genes identified to be susceptible to variants in PID may also be subject to heritable epigenetic modifications which could lead to the same or similar symptoms, acting as a further coefficient value when calculating the potential number of disorders, including those disorders affected by splicing, which can cause PID (Zhu et al., 2018).

### RNA in Diagnostics

RNA investigation technology and its literature has experienced great leaps forward in recent years in terms of technological advancement and cost reduction (Muir et al., 2016). RNA sequencing is now largely replacing microarrays as the most used quantitative method of mapping gene expression profiles (Lowe et al., 2017). The transcriptome—or RNA expression profile—of a given tissue can give unparalleled insights into the elegant inner workings of the cell. Through capture of all internal RNA species, it characterizes the cellular gene transcription architecture and can deliver an instantaneous picture of environment–cell interaction or response program (Lowe et al., 2017; Wirka et al., 2018).

A range of technologies exist for conducting RNA sequencing, each with its own strengths and limitations. Long-read sequencing provides reliable structural information, but can have suboptimal reliability in base calling (Feng et al., 2015) or is more expensive for high-throughput analysis (Rhoads and Au, 2015). Short-read NGS RNASeq involves sonication or enzymatic degradation of RNA into smaller fragments, selection of fragments using one of a number of methods, cDNA synthesis, the construction of a library, and subsequent sequencing followed by realignment (Kukurba and Montgomery, 2015). This technology has been the currently favored approach for high-throughput analysis.

Currently, this technology generates a mixture of both quantitative and qualitative analysis opportunities of RNA species: qualitative transcriptome profiling outcomes include identification of sequence variants at the level of the genome (Neums et al., 2017), somatic cell mosaics, non-canonical splice variants, occurring either due to *cis*- or *trans*-acting factor aberrations (Ward and Cooper, 2010). Quantitative outcomes of transcriptional profiling include differentially expressed genes, alternative splicing events, and allele-specific expression quantification (Kukurba and Montgomery, 2015). Previous studies have demonstrated that when compared with large control datasets, identification of expression outliers in peripheral whole blood can contribute to the detection of disease-causing variants (Zeng et al., 2015; Zhao et al., 2016). As well as gene expression levels, perturbations in the relative abundance of specific isoforms is a driving force in the genesis of many diseases (Chen et al., 2010; Kim et al., 2018) as isoforms can have differential function (Takeda et al., 2010), or, in some cases, can be antagonistic (Eshel et al., 2008). Through RNASeq or exon junction spanning probe-based capture, changes in isoform balance can also be resolved. The sensitivity suitability of RNASeq in transcriptomic investigation and splicing was demonstrated in mouse and human models and has enabled the discovery of ∼7,600 novel isoforms in mouse immune cells (Ergun et al., 2013) and detected 100,000 splicing events with at least moderate abundance (Pan et al., 2008).

Transcriptome profiling can also give insights into control mechanisms exhibited by the non-coding RNA species, such as long non-coding RNA (lncRNA) and microRNA (miRNA), the significance of which is continually being elucidated in the molecular pathology of disease (Kramer et al., 2015; DiStefano, 2018). Indeed, such examples exist in PID; miR-6891-5p accumulation is demonstrated to contribute to selective IgA deficiency, the most common form of PID (Chitnis et al., 2017). Thanks to the increasing ability of technology and steady reduction in costs, we are also able to cast a winder net. Through RNASeq-based investigation, instead of concentrating on *a priori*, system-specific gene panels that many studies target, it is possible to examine all the mRNA species destined for translation. Through these hypothesis-free methods, it is possible to create profiles of normal transcription and disease transcription in a tissue-specific manner (Gonorazky et al., 2019). Subsequent use of follow-up analysis tools can be used to generate filtering process for causal variants or for biomarker identification (Han and Jiang, 2014). It is also possible to quantify the relative expression of those genes coding for the splice factors themselves, which can directly bring about pathological processes specific to PID, such as those observed in Roifman’s syndrome, mentioned earlier (Merico et al., 2015; Heremans et al., 2018).

Microfluidic technology adaptations have allowed the development of robust, single-cell transcriptomic profiling (Kimmerling et al., 2016). In combination with NGS-based technologies, the single-cell technology provides a method for profiling the transcriptomes of individual cells, giving unparalleled insights into the heterogeneity of cell populations and their transcriptional profiles (Hwang et al., 2018). Adaptations such as the SMART-seq2 or fluidigm C1 library preparation methods also now allow the production of full-length cDNAs, giving transcript isoform-level resolution. However, these methods do not yet allow multiplexing, massively increasing overall costs and labor in large cohorts (See et al., 2018). The ability to profile the entire transcriptome of a peripheral blood mononuclear cell (PBMC) culture individually would give a dramatically increased ability to understand the cell–cell interactions taking place in an immune challenge, and this approach could be utilized in those patients suspected to be genetic mosaics.

## Discussion

**Figure 2:**
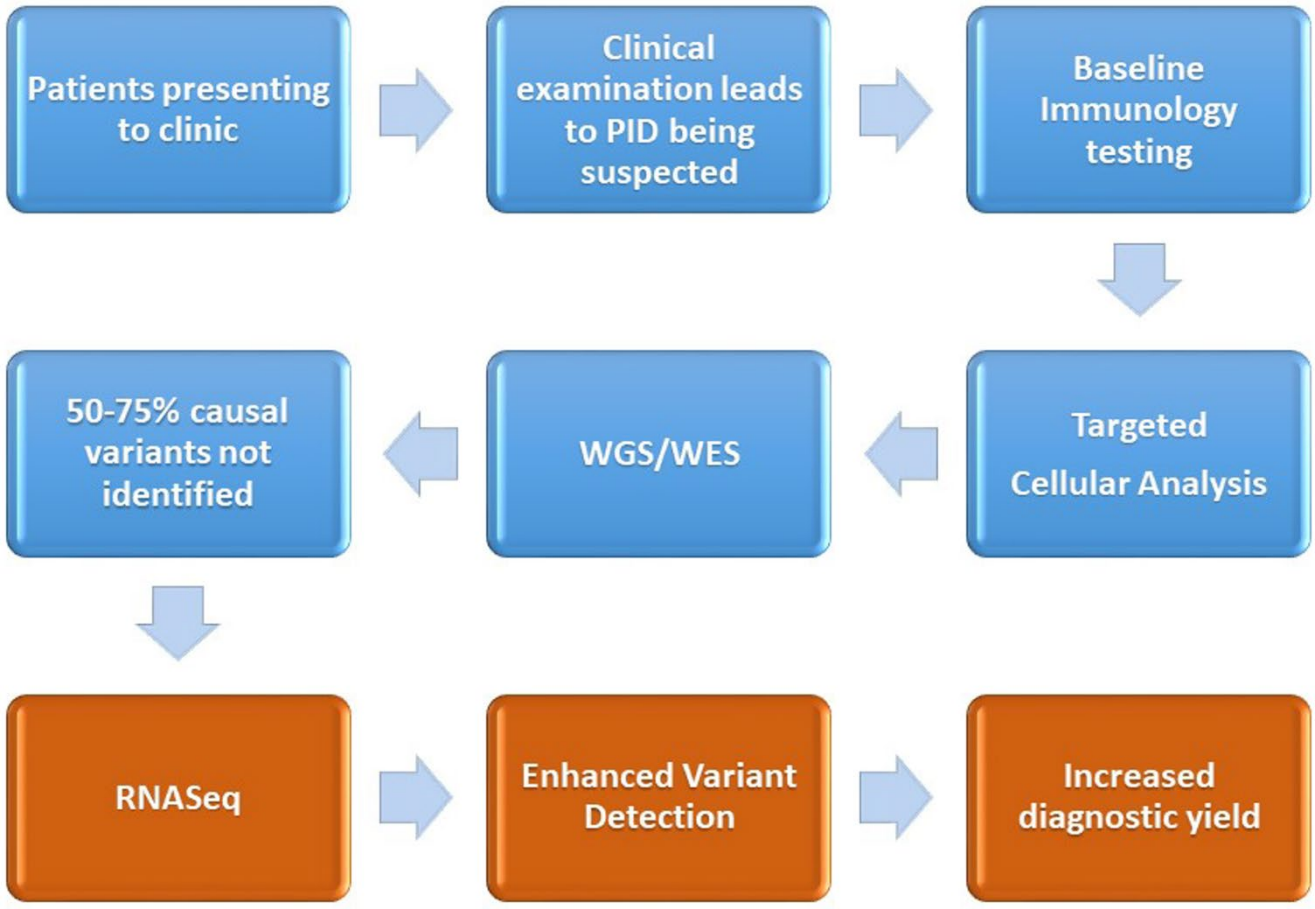
Demonstration of the current diagnostic pathway (*blue*) for the majority of patients after a diagnosis of PID is suspected. We also outline the proposed intervention point of RNASeq and the associated enhanced variant detection (coming about through assessment of differential expression, changes to alternative splicing) and the increased diagnostic yield.

The early and accurate diagnosis of primary immunodeficiencies is important to ensure the attainment of positive patient outcomes, through minimizing the time to diagnosis, identifying molecular pathways for targeted therapy, and reducing the economic cost of ill health or inappropriate treatment options. Diagnosis of the disorders remains difficult due to clinical challenges in identifying the presence of a primary immune system disorder, stratifying the phenotype to a myriad of overlapping candidate genes and then the laborious task of variant filtering, interpretation, and lack of knowledge pertaining to variants, especially those residing in the non-coding segments of the DNA. Functional validation of a candidate variant is currently undertaken with protein-based *ex vivo* tests, which are difficult to standardize and mostly available in research laboratories. RNA profiling to identify alternative splicing, gene expression-level variation monoallelic expression may contribute a further insight into candidate variants derived from proband or family-based WES/WGS sequencing results. We propose the introduction of RNASeq-based analysis for patients who have a clinical presentation of PID, but who, despite normal baseline immune testing, cellular analysis, and having undergone WES/WGS remain undiagnosed (see **Figure 2**).

RNASeq is an emerging technology which, when combined with WES/WGS, provides unprecedented insights into differential gene expression, splicing activity, and allelic specific expression and can inform regarding other phenomena such as genetic mosaicism. However, RNASeq remains relatively novel as a diagnostic testing tool in rare diseases and the control datasets and cellular contributions to complex tissue profiles (i.e., whole blood) will require further dissection.

Utilizing candidate gene lists and large control datasets for comparison enhances the power of the transcriptional profiling through RNASeq and improves resolution for differential gene expression. Existing projects have developed these datasets for whole blood and immune cells, which provide a starting point for the interrogation of clinical samples for diagnostic research.

Immune responses to pathogenic challenges are exceptionally variable, and the variability in these responses is not easily elucidated. Environmental influences such as age, sex, seasonality, nutrition, and lifestyle all have effects on the specific response profile exhibited by individuals (Piasecka et al., 2018). These factors that influence responses can have a greater degree of significance in specific cell types. CD8^+^ T cells, for example, show a high degree of heterogeneity in the context of temporal changes through the life course of the individual, and CD4^+^ T cells and monocytes are heavily influenced by sex (Piasecka et al., 2018). It is therefore useful to be able to discern transcripts from different cell types within a culture. Utilizing flow cytometry to separate cell types or utilizing single-cell RNASeq is becoming an attractive option.

In order to assess the impact of genomic variation on the unstimulated immune system, the normal immune response and the immune-deficient responses, it is important to experimentally “tune out” the variations in signal arising from environmental factors. It has been established that a high degree of the cellular variation in CD8^+^ cell populations can be attributed to environmental factors, which makes them a poor model for genetic variant impact. CD4^+^ T cells display a large degree of heritability in these assays, and as such should provide a good level of transcriptomic heritability also. This will allow for clearer elucidation of the effects of variants on differential gene expression (Brodin et al., 2015).

The immune system’s response to pathogen-based challenges is highly dynamic, and observing this response is more informative when identifying impaired response (Duffy et al., 2017). Indeed, it has been shown in innate immune system studies that the effects on differential expression of some variants can only be observed in a dynamic fashion (Fairfax et al., 2014; Lee et al., 2014). Co-culture of PBMCs provides a greater insight into activation pathways as it allows for the cell–cell communication response programs and produces similar results in terms of ranked gene expression response networks, with a few notable exceptions (Duffy et al., 2017). Studies of dynamic immune responses to challenges, in concert with machine learning, can be used to identify small groups of stimulation pathway-specific genes (Urrutia et al., 2016). Comparing the expression profiles of these genes in healthy cohorts with PID patients can potentially be utilized to identify candidate genes, which may then harbor a disease-causing variant or indicate some anomaly in the pathway for further investigation.

The transcriptomic landscape provides an excellent opportunity for advancement of diagnostic yield, and transcriptional profiling is already being utilized across a range of disorders to help build a “molecular fingerprint” of disease and better inform variant-filtering processes. The immunology community has made a case for PID diagnosis to be supported using transcriptional profiling using whole-transcriptome sequencing (Moens et al., 2014), and these are being answered with examples in primary immunodeficiency cases such as Dock8 CID, GATA2 deficiency, and X-linked reticulate pigmentary disorder (XLPDR) (Hsu et al., 2013; Khan et al., 2016; Starokadomskyy et al., 2016). Over the coming years, an extended diagnostic approach to PID testing may develop that builds on a clinical module of phenotype, family history, and baseline immunological testing. This will be complemented by a DNA module of coding and non-coding variant analysis, utilizing sophisticated bioinformatic pipelines to prioritize candidate genetic variants of new loci that would be consistent with the clinical phenotype and family segregation. These candidate variants for monogenic disease may then be functionally interrogated *via* RNAseq for an influence within the gene itself and possibly the network within which it operates. In parallel, functional testing of candidate genes through protein-based assays may be undertaken to characterize the impact of a putative monogenic pathogenic variant within a reductionist model at the protein level. The sharing of these modular assessments across the international community will incrementally improve the standardized analysis of novel variants that will continue to grow over the next few years.

## Author Contributions

DB and AW devised and planned the project and co-supervised JL. JL wrote the manuscript. DB and AW reviewed, edited and contributed to the manuscript.

## Funding

This work was funded by a NIHR research (grant number: NIHR RP2026-07-011) professorship to DB.

## Conflict of Interest

The authors declare that the research was conducted in the absence of any commercial or financial relationships that could be construed as a potential conflict of interest.

## Glossary of Terms

NGS (next-generation sequencing): Next-generation sequencing describes the massively parallel, high-throughput modern evolution of sequencing.
WES (whole-exome sequencing): Whole-exome sequencing is the process of reading and recording the nucleotide sequence of all the protein coding regions of a subject’s genome (termed the exome).
WGS (whole-genome sequencing): Whole-genome sequencing is the process of reading and recording the nucleotide sequence of the entirety of a subject’s genome.
RNASeq (RNA sequencing): RNA sequencing is the process of capturing and sequencing the available RNA from a tissue or cell. This can include all transcribed RNAs, or just the messenger RNA, depending on the capture method.
Short-read sequencing: Short-read sequencing is the process of sequencing a pre-fragmented library of nucleic acid, typically 75–300 bp in size.
Long-read sequencing: Long-read sequencing in the process of sequencing full-length nucleic acid fragments.
